# Development of a Formulation and In Vitro Evaluation of a Pulmonary Drug Delivery System for a Novel Janus Kinase (JAK) Inhibitor, CPL409116

**DOI:** 10.3390/pharmaceutics16091157

**Published:** 2024-08-31

**Authors:** Aleksandra Rzewińska, Jakub Szlęk, Damian Dąbrowski, Ewelina Juszczyk, Katarzyna Mróz, Heikki Räikkönen, Mia Siven, Maciej Wieczorek, Przemysław Dorożyński

**Affiliations:** 1 Finished Dosage Forms Department, Research and Development Center, Celon Pharma S.A., Marymoncka 15, 05-052 Kazuń Nowy, Poland; ewelina.juszczyk@celonpharma.com (E.J.); k.mroz@celonpharma.com (K.M.); 2 Department of Drug Technology and Pharmaceutical Biotechnology, Faculty of Pharmacy, Medical University of Warsaw, Banacha 1, 02-097 Warszawa, Poland; przemyslaw.dorozynski@wum.edu.pl; 3 Chair and Department of Pharmaceutical Technology and Biopharmaceutics, Faculty of Pharmacy, Jagiellonian University Medical College, Medyczna 9, 30-688 Kraków, Poland; j.szlek@uj.edu.pl; 4 Chair of Analytical Chemistry, Faculty of Chemistry, Warsaw University of Technology, Noakowskiego 3, 00-664 Warszawa, Poland; damian.dabrowski@pw.edu.pl; 5 Faculty of Pharmacy, University of Helsinki, Viikinkaari 5, 00014 Helsinki, Finland; heikki.raikkonen@helsinki.fi; 6 Division of Pharmaceutical Chemistry and Technology, Faculty of Pharmacy, University of Helsinki, Viikinkaari 5 E, 00014 Helsinki, Finland; mia.siven@helsinki.fi; 7 Helsinki Institute of Sustainability Science HELSUS, University of Helsinki, 00014 Helsinki, Finland; 8 Research and Development Center, Celon Pharma S.A., Marymoncka 15, 05-052 Kazuń Nowy, Poland; maciej.wieczorek@celonpharma.com; 9 Department of Inorganic and Analytical Chemistry, Faculty of Pharmacy, Jagiellonian University Medical College, Medyczna 9, 30-688 Kraków, Poland

**Keywords:** Janus kinase (JAK) inhibitor, pulmonary drug delivery, spray drying (SD), micronization, jet milling (JM)

## Abstract

The pursuit of targeted therapies for cytokine-dependent diseases has led to the discovery of Janus kinase (JAK) inhibitors, a promising class of drugs. Among them, CPL409116, a selective dual JAK and rho-associated protein kinase inhibitor (ROCK), has demonstrated potential for treating conditions such as pulmonary fibrosis exacerbated by the COVID-19 pandemic. This study investigated the feasibility of delivering CPL409116 via inhalation, with the aim of minimizing the systemic adverse effects associated with oral administration. Two micronization methods, jet milling and spray drying, were assessed for CPL409116, with spray drying chosen for its ability to produce an amorphous form of the compound. Moreover, parameters such as the mixing energy, drug load, and force control agent significantly influenced the fine particle fraction (FPF), a critical parameter for pulmonary drug delivery. This study provides insights into optimizing the formulation parameters to enhance the delivery efficiency of CPL409116 to the lungs, offering potential for improved therapeutic outcomes in cytokine-dependent pulmonary diseases.

## 1. Introduction

The mammalian Janus kinase (JAK) family consists of four receptor-associated tyrosine kinases: JAK1, JAK2, JAK3, and tyrosine kinase 2 (TYK2). These kinases play a crucial role in the signal transduction cascade of cytokine-binding receptors [1,2,3]. Among the various families of cytokine-binding receptors, such as the interleukin (IL)-1 receptor superfamily, the IL-17 receptor superfamily, the receptor tyrosine kinase superfamily, the transforming growth factor receptor superfamily, the G protein-coupled receptor superfamily, and the tumor necrosis factor (TNF) receptor family, JAKs specifically modulate only the activity of the type I and type II cytokine receptor superfamily [4]. Numerous JAK-dependent cytokines contribute to the manifestation of symptoms in autoimmune or inflammatory diseases, such as IL-6 in rheumatoid arthritis [5] and IL-12 and IL-23 in inflammatory bowel disease and psoriasis [6]. As a result, the targeting of JAKs has emerged as a therapeutic strategy for the treatment of cytokine-dependent diseases.

The first JAK inhibitor approved by the FDA in 2011 was ruxolitinib, which is orally administered for the treatment of myelofibrosis and graft-versus-host disease [7,8]. Additionally, topical ruxolitinib is used to treat atopic dermatitis and vitiligo [3]. Subsequently, many oral JAK inhibitors have been released for indications, such as rheumatoid arthritis [1,9], inflammatory bowel diseases (ulcerative colitis [10]), and alopecia areata [11]. While the oral administration of JAK inhibitors is convenient for patients, it is also associated with systemic adverse effects, such as cytopenia, immunosuppression, increased susceptibility to infections, and the reactivation of herpes zoster [12,13]. These effects may be acceptable in the treatment of conditions such as oncological diseases, where the therapeutic benefits outweigh the side effects. However, for chronic diseases, minimizing significant adverse effects is crucial.

Diseases such as asthma and post-COVID-19 pulmonary fibrosis (PC19-PF [14]) are closely linked with inflammation and immunopathological responses. The direct administration of medication to the lungs could provide the rapid onset of action and increased effectiveness through lower doses, resulting in reduced systemic adverse effects [15,16]. JAK inhibitors are being widely tested in phase I clinical trials for people with mild asthma [15]. To the best of our knowledge, Astra Zeneca’s compound AZD0449, developed as a dry powder inhaler (DPI), is the only one to have reached the first phase of clinical trials in this form [17]. There are extensive data from animal testing where JAK inhibitors in liquid form were administered intratracheally [15,18].

This investigation focused on the compound CPL409116, which was selected from the Celon Pharma company library as a potent, selective JAK/ROCK inhibitor, demonstrating a greater selectivity for JAK1 and JAK3 compared to JAK2. Additionally, CPL409116 effectively suppressed ROCK1 and ROCK2 kinases [19]. In vitro studies have shown that CPL409116 effectively reduces the expression of ASMAs (anti-smooth muscle antibodies), which are increased in fibrosis processes. Furthermore, CPL409116 was shown to decrease the level of MLC phosphorylation, indicating the inhibition of fibrosis. In light of these findings, particularly following the SARS-CoV-2 pandemic, which resulted in lung fibrosis in many patients, an investigation was initiated to assess the feasibility of delivering this compound directly to the lungs.

There is a knowledge gap regarding the development of dry powder for inhalation containing JAK inhibitors as an active substance. Further research is needed to develop a safe and effective inhaled formulation that will reduce the risk of side effects. From the authors’ perspective, recognizing the limitations of the active pharmaceutical ingredient (API) and exploring various technological approaches in the development process is crucial. Addressing these aspects may reduce costs and save valuable time to market.

The main goal of this investigation was to assess the feasibility of administering the JAK inhibitor CPL409116, developed by Celon Pharma S.A., in a carrier-based dry powder formulation via the inhalation route. The most important engineering factors affecting the performance of these formulations include API processing, carrier modifications, the mixer type, and the mixing parameters (time, speed) for blending the API with lactose-based carrier particles, as well as the inclusion of ternary components such as force control agents or additional fines [20,21]. This research, representing the first stage in the development of a dry powder inhalation formulation containing the innovative CPL409116 molecule, involved an extensive investigation into two approaches for CPL409116 micronization. It also included an analysis of the quantitative and qualitative composition of the developed formulation, focusing on the drug load and ternary component.

Additionally, this investigation aimed to identify, through mathematical modeling, the key formulation development factors that have the greatest impact on achieving a high fine particle fraction (FPF), which is needed for pulmonary delivery.

## 2. Materials and Methods

### 2.1. Characteristics of Investigated Materials

**Figure 1:**
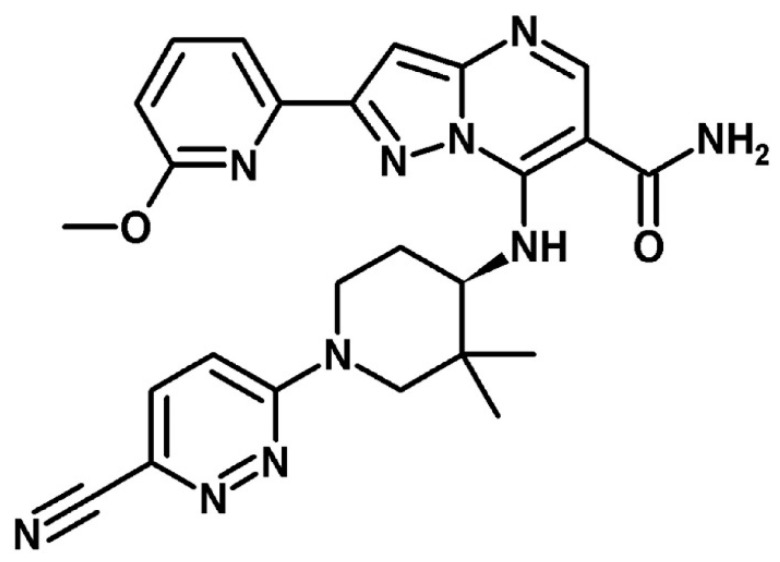
Chemical structure of CPL409116 ((R)-7-((1-(6-cyanopyridazin-3-yl)-3,3-dimethylpiperidin-4-yl)amino)-2-(6-methoxypyridin-2-yl)pyrazolo [1,5-a]pyrimidine-6-carboxamide).

CPL409116, the active substance, was synthetized at the Łukasiewicz Research Network—Industrial Chemistry Institute (Warsaw, Poland). The chemical structure is presented in Figure 1.

Lactose from DFE Pharma, Germany, was used to produce carrier-based blends. The density of the lactose monohydrate carrier particles was 1.529 g/cm^3^. Magnesium stearate (MgSt) LIGAMED MF-2-V-BI (Peter Greven Gmbh and Co. KG, Bad Münstereifel, Germany) was used as a force control agent. 

### 2.2. Technologies for API Processing

#### 2.2.1. Jet Milling

The jet-milling process was conducted using a Spiral Jet-Mill MC150 (Micro-Macinazione, Lugano, Switzerland). This procedure utilized unprocessed CPL409116 with an initial crystal size range of 500–1500 µm. The dosing pressure was set to 8.0 bar, the rate was 266 g per hour, and the milling pressure was 1.5 bar. The micronized material was conditioned for a period of 24 h and then transferred into an antistatic polyethylene bag for subsequent analysis and storage.

#### 2.2.2. Spray Drying

Spray drying was performed in a Mini Spray Dryer B-290 (Buchi Labortechnik AG, Flawil, Switzerland) equipped with a 0.7 mm nozzle in a closed, inner loop mode with nitrogen. The liquid feedstock of CPL409116 for spray drying was prepared by dissolving CPL409116 in pure acetone (Sigma Aldrich, Merck Group, Darmstadt, Germany) to a final concentration of 17.9 g·L^−1^ (*w*/*v*).

The atomization gas flow and the pump speed were kept fixed throughout the whole process and were set to 6.83 L/min and 0.0025 L/min, respectively.

During drying, the dry particles were separated from the airflow using a Büchi standard cyclone. The material collected after the process was transferred to glass vials for further analysis and storage. All the powder handling and sample preparation procedures for the analysis were carried out at a temperature of 22 ± 1 °C and a relative humidity of 23 ± 3%, as described elsewhere [22].

### 2.3. Physicochemical Analysis of the API

#### 2.3.1. Particle Size Distribution (PSD)

The particle size of the micronized CPL409116 was measured using a laser light-scattering technique with a dry dispersion particle size analyzer (RODOS/HELOS/R1 with Aspiros, Sympatec, Clausthal-Zellerfeld, Germany). An amount of 30 mg of micronized powder was weighted and transferred directly into the Aspiros vial, which fed the sample at a speed of 15 mm/s into the RODOS, operating with a dispersion pressure set to 3.0 bar and a vacuum of 0.0527 bar. The powders were introduced at an optical concentration of approximately 5%, and the data were collected over a measurement duration of up to 5 s. Subsequently, the mean particle sizes based on the volume diameter and the standard deviations were calculated from three separate analyses.

The measurements were characterized by D [v. 0.1] (10% of the volume distribution was below this value), D [v. 0.5] (the volume median, defined as the diameter where half of the population fell below this value), and D [v. 0.9] (90% of the volume distribution was below this value). In addition, the SPAN value was calculated according to Equation (1):

(1) $$\mathrm{Span}=\frac{D\left[0.9\right]-D\left[0.1\right]}{D\left[0.5\right]}.$$

The SPAN value characterizes the width of the particle size distribution: the higher the SPAN, the wider the distribution [23].

#### 2.3.2. Particle Morphology

This study was performed using a Morphologi G3s automatic microscopic analyzer under diagonal light (Malvern Panalytical, Worcestershire, UK). Microscopic imaging of a sample volume of 1 mm^3^ was carried out after air dispersion in a sample dispersion unit (SDU), with the dispersal pressure set to 5.0 bar, a dispersion time of 60 ms, and a settling time of 100 s. The lenses used had magnifications of 10×, 20×, and 50×. The scan area was set to approximately 450 mm^3^. The morphological output parameters for the individual particles comprised the length, width, volume, diameter, and shape (aspect ratio, circularity, and elongation).

#### 2.3.3. Scanning Electron Microscopy (SEM)

Solid particles were scattered onto double-sided tape and sputtered with platinum for 25 s (5 nm) at a pressure of 0.3 bar in a Turbo-Pumped Sputter Coater before they were analyzed using SEM.

SEM images of the selected samples were made on a Quanta 250 Field Emission Gun Scanning Electron Microscope (FEI, Hillsboro, OR, USA) at an accelerating voltage of 5 kV.

#### 2.3.4. X-ray Powder Diffraction (XRPD)

The X-ray powder diffraction (XRPD) patterns of the CPL409116 samples were recorded using X-ray powder diffractometry (Malvern Panalytical, UK) with Cu Kα radiation (λ = 1.54 Å). Each sample was prepared by placing the powder into aluminum holders and scanned over the 5–40° angular range (2θ) at a rate of 0.1°/s using a Cu radiation source with a wavelength of 1.54 Å, operating at 40 kV and 7.5 mA.

#### 2.3.5. Intrinsic Dissolution

The intrinsic dissolution test was performed using the USP II apparatus with a rotating disk. The API was pressed at a pressure of 3 tons to an area of 0.5 cm^2^. The dissolution medium was 900 mL of 0.1 M HCl with 0.5% of SDS, the rotation of the disc was 100 rpm, and the temperature of the medium was 37 °C. Preliminary studies showed that CPL409116 was freely soluble in the chosen medium. Samples were collected at time points of 0.5, 1, 2, 3, 4, and 5 h, and then they were filtered through a 0.45 µm cellulose syringe filter. The concentrations were assessed using reversed-phase high-performance liquid chromatography (RP-HPLC) with UV detection at 258 nm, as described in Section 2.5.

### 2.4. Preparation of Blends and Encapsulation

Two types of blenders were employed for the blend preparation, depending on the required mixing energy: a high-shear blender (1L TRV, GEA) and a V-shear blender (Laskus, Karczew, Poland). The mixing energy (ME) was determined according to Equation (2) [24]:

(2) $$ME=8\pi^{3}\cdot m\cdot {\left(\frac{rpm}{60}\right)}^{3}\cdot r^{2}\cdot t,$$

where m is the carrier mass; rpm denotes revolutions per minute; r is the blender radius; and t is the mixing time. 

The blend was encapsulated on a DrumLab encapsulation machine (Harro Hoffliger, Allmersbach im Tal, Germany) into inhalation-grade hydroxypropylmethylcellulose (HPMC) capsules with a size of 3 (Quali-VI^®^, Qualicaps, Madrid, Spain). The capsules were combined with a low-resistance version of the RS01 DPI from Plastiape, S.p.A., Osnago, Italy.

The encapsulation process was conducted with a drum system, allowing for net masses of approximately 25 mg, depending on the tested formulation. The process parameters directly affecting the powder mass uniformity included the blow-out pressure, which was maintained within the range of 2.0 to 1.5 bar, depending on the blend rheology, as well as the vacuum pressure setting, which was set within the range of −3.0 to −0.5 bar in the machine control software.

### 2.5. Aerosol Performance Testing

The aerodynamic particle size distribution (APSD) test was conducted using a Next Generation Impactor (NGI, Copley Scientific Limited, Nottingham, UK) fitted with a USP induction port. The analysis was carried out by following the guidelines specified in USP 〈601〉: “Aerosols: Aerodynamic Size Distribution, Apparatus six for Dry Powder Inhalers” and Ph. Eur. 2.9.18: “Preparations for Inhalation: Aerodynamic Assessment of Fine Particles; Apparatus E”. APSD assessments were performed under the conditions of a 4 L volume and a 4 kPa pressure drop, which was equivalent to an approximate volumetric flow rate of 100 L/min. 

The powder collected from stages 1–7 (using a micro-orifice collector, MOC), the mouthpiece, and the pre-separator from the NGI were rinsed with HPLC solvent. The drug content was subsequently analyzed using RP-HPLC (described in the next paragraph). Based on the data obtained from drug deposition, the FPF was calculated using CITDAS version 3.10, a data-processing software provided by Copley Scientific, Nottingham, UK. 

The aerosol test samples were analyzed using RP-HPLC, with spectrophotometric detection at a wavelength of 258 nm. The samples were analyzed with the Agilent 1200, Agilent 1260 Infinity II, Agilent 1290 Infinity II (Wilmington, DE, USA), or Shimadzu Prominence LC-2030C 3D Plus systems (Canby, OR, USA). The separation was achieved with an Agilent Zorbax Eclipse Plus C18, 3.0 × 50 mm, 1.8 μm column (P/N 959941-302) using a gradient method (0 min—57% B; 7—57%; 9—90%; 9.5—57%; 12—57%). Solvent A comprised water with 0.1% formic acid; solvent B was methanol. CPL409116 was quantified using a single-point calibration with a reference standard. The samples were extracted with water/acetonitrile in a ratio of 1:1 (*v*/*v*).

This method underwent in-house validation. The linearity represented by the R2 value of the calibration curve within the range of 10% to 160% of the target API mass in the capsule was determined to be 0.9999. The LoD (limit of detection) and LoQ (limit of quantification) were found to be 2.05 and 6.22 µg/mL, respectively. The precision, evaluated as a %RSD of the retention time and peak area, was 0.1% and 0.3%, respectively, with an API peak symmetry factor of 1.04. The accuracy, calculated as the mean recovery from a spiked placebo sample, was 100.6%.

In addition to APSD, the delivered dose (DD) was also determined in vitro using the dosage unit sampling apparatus (DUSA, Copley Scientific Limited, Nottingham, UK) and the RP-HPLC method described above. The same RP-HPLC method was utilized to assess the content uniformity. Ten samples were taken with a sample thief at three different locations within the blender, following a sampling scheme of three from the top, four from the middle, and three from the bottom.

### 2.6. Stability Studies

Stability testing was conducted for three months under long-term (25 °C; 60% RH) and accelerated (40 °C; 75% RH) conditions for two blends containing 7.5% and 30% of API, respectively. The studies were conducted in stability chambers (BINDER KBF 240). 

The analysis of related substances was conducted using HPLC with spectrophotometric detection at a wavelength of 297 nm. Separation was achieved with an Agilent Zorbax Eclipse Plus Phenyl-Hexyl 3.0 × 150 mm and a 3.5 µm column using a gradient method (0 min—15% B; 10—34.5%; 24—57%; 27—90%; 30—90%; 31.2—15%). Solvent A was 0.1% TFA/H2O and solvent B was 0.1% TFA/ACN. CPL409116 was quantified using a single-point calibration with a reference standard. The samples were extracted with water/acetonitrile/TFA in a ratio of 75:25:0.1 (*v*/*v*). 

The main parameters determined during the validation of this method were the resolution, which was calculated between the two closest specified impurities and was found to be 5.71, and the LoD and LoQ of the API, which were 0.025 and 0.063 µg/mL, respectively.

### 2.7. Implementing Symbolic Regression

To understand the relationships between the process parameters and the FPF, we employed a workflow with the following steps. First, we applied a genetic programming method, specifically symbolic regression, using PySR. We chose symbolic regression because it offers both interpretability and suitability for capturing complex patterns within the data. Moreover, symbolic regression allows relationships to be expressed in a human-understandable form, such as a mathematical equation, which is easy to interpret. Additionally, symbolic regression has been successfully applied in similar research, demonstrating its effectiveness in extracting meaningful insights from data.

The model was developed using experimental data. Twelve records of the data (twelve formulations) were split into training (ten records) and testing (two records) datasets. To assess the robustness of the model, enhanced data were generated by adding Gaussian noise (standard deviation = 10%, multiplied 10 times) to the data, resulting in a validation dataset consisting of 120 records.

For selecting the best model, we employed the root-mean-squared error (RMSE) across multiple stages of the modeling process, including training, testing, and validation, to ensure consistency and robustness in the model performance assessment. We aimed to minimize the RMSE across all the datasets while avoiding overfitting. A model with consistently low RMSE values across all the datasets was considered the best candidate, indicating its effectiveness in capturing the underlying relationships between the process parameters and the FPF while maintaining a robust performance across different datasets.

Next, we used the best model obtained for a SHAP (Shapley additive explanations) analysis. A SHAP analysis is a method used to explain the output of machine learning models by attributing the contribution of each feature to the model’s prediction. It is based on cooperative game theory and calculates the importance of each feature by considering its impact on the prediction across all possible combinations of features. SHAP values were computed for each feature, indicating how much each feature contributed to the model’s prediction of the FPF for individual data points. This allowed us to identify which process parameters had the most significant influence on the final particle fraction and to understand the direction and magnitude of their effects. 

## 3. Results and Discussion

In this study, the feasibility of developing a dry powder inhalation formulation containing a selective JAK/ROCK inhibitor as the active substance was evaluated. After observing the antifibrotic properties of the compound CPL409116 in internal in vitro tests, it was decided to investigate the feasibility of developing a dry powder formulation for inhalation containing CPL409116 as the active substance.

The development process was divided into two main parts. The first part involved selecting an appropriate technological process for micronizing the active substance. The second part encompassed the preparation of a carrier-based formulation, the assessment of its aerodynamic properties using the NGI, and the determination of the factors that influence the critical parameter ensuring pulmonary delivery—the FPF.

### 3.1. Tailoring the API Structure

The first stage of this work aimed to choose a technological process capable of producing API particles suitable for inhalation administration. As reported by Shety et al., micron-sized particles for inhalation are commonly obtained through jet milling [25]. However, for high-dose inhalable products, particle engineering approaches such as spray drying (SD) are employed [26,27]. Spray-drying technology minimizes the cohesion of the particles while enhancing their dispersibility and improving the delivery of the API [28].

According to the literature, spray drying is most frequently used for the development of carrier-free platforms (without lactose monohydrate as a carrier). However, there are studies indicating the possibility of formulating carrier-based platforms where only the API is spray-dried [29]. 

CPL409116 is a compound with a low solubility in solvents suitable for lactose, mannitol, or trehalose, which are used to create suspensions or solutions for spray drying to form carrier-free platforms. Therefore, it was decided to adopt an approach involving the spray drying of the API alone. Apart from reducing the cohesion between molecules, the process of spray drying can yield an amorphous form of the compound, which is reported to enhance its solubility. Due to these factors, both spray drying and jet milling were employed as the micronization techniques for CPL409116.

The jet-milling method micronizes particles by employing high-pressure, compressed gas to create high-velocity collisions between particles of the raw material. In the spray-drying approach, the API is dissolved or suspended in the solvent, and the prepared solution or suspension is spray-dried using suitable parameters.

#### 3.1.1. Particle Size Distribution of CPL409116

It is assumed that the particles aimed at reaching the alveolar region in the lung should have an aerodynamic diameter within the range of 1–5 µm [21,30,31]. Finer particles are more likely to be exhaled, while coarser particles may be swallowed or may collide with the walls of the upper respiratory tract [32]. Therefore, the particle size of the active substance is a critical parameter in the evaluation of carrier-based dry powder formulations for inhalation [33].

**Figure 2:**
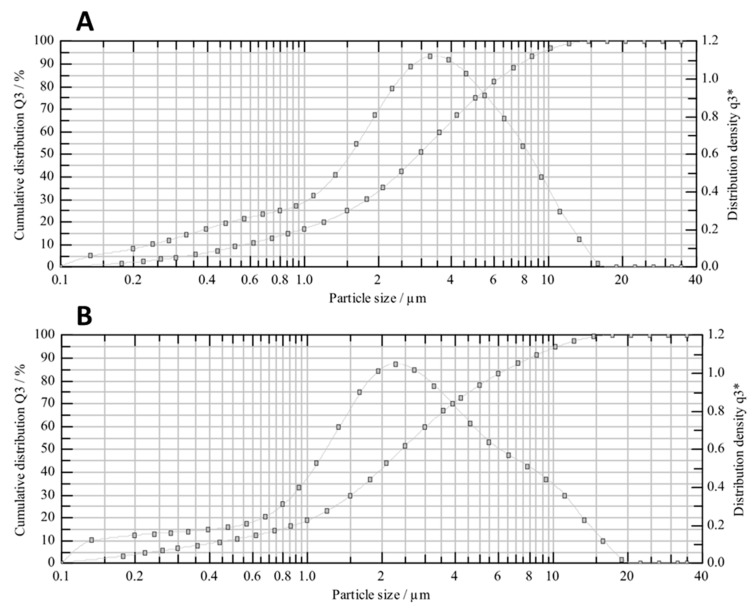
Particle size distribution profile of jet-milled (**A**) and spray-dried (**B**) CPL409116, presented as cumulative distribution and distribution density.

The particle size distribution (PSD) results obtained from both processes (jet milling and spray drying) showed that both methods enabled the production of a powder mainly composed of particles with a diameter of less than 10 μm. In the jet-milled API, 90% of the particles had a diameter smaller than 7.75 μm (Figure 2A), with a SPAN value of 2.42. After spray drying, 90% of the particles had a diameter smaller than 8.13 μm, which was 5% higher with respect to jet milling. However, the SPAN value increased by 30% to 3.14. This can be explained by the fact that the jet-milled compound showed a narrower distribution than the spray-dried one (Figure 2B).

A summary of the PSD results for both processes is provided in Table 1, including the volume at the 10th, 50th, and 90th percentiles, as well as the SPAN value.

#### 3.1.2. Morphology of CPL409116

**Figure 3:**
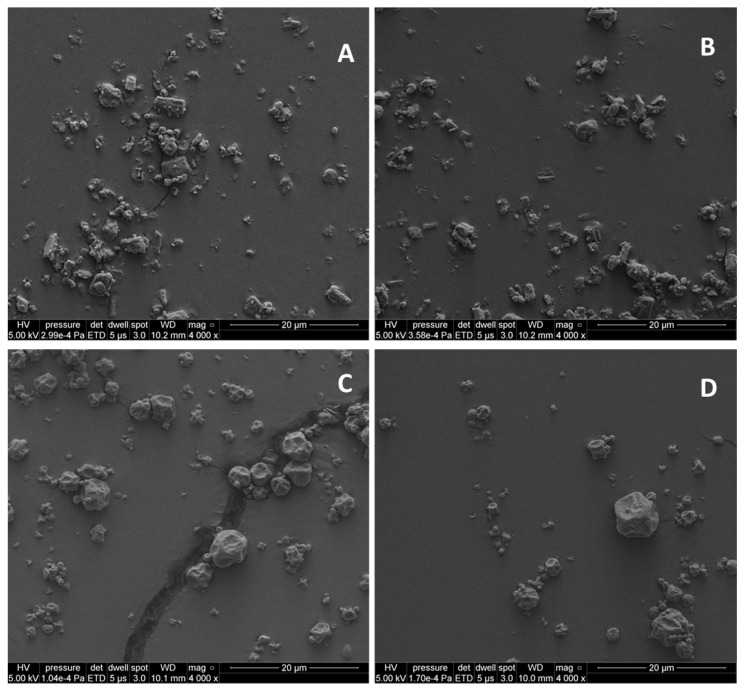
SEM images of jet-milled (**A**,**B**) and spray-dried (**C**,**D**) CPL409116 particles.

In addition to the particle size, the morphology of the particles—the shape, length, width, and surface area—is crucial to the performance of the product [27,34]. These parameters were determined using the Morphologi G3 technique and were further supported by the qualitative visual examination of scanning electron micrographs. The SEM results are presented in Figure 3 and show that, despite a similar size distribution, the morphology of the particles after spray drying and jet milling was different. 

The CPL409116 particles after jet milling were elongated, while the spray-dried particles were spherical with a concave surface, which is typical of spray drying with acetone [35].

#### 3.1.3. Solid-State Form of CPL409116

During the development of an inhaled drug product, it is crucial to characterize the tested material in terms of its crystalline or amorphous form. The transition from one form to another may directly impact the therapeutic activity of the compound or indirectly affect factors such as the stability of the drug product and its efficacy [36]. 

**Figure 4:**
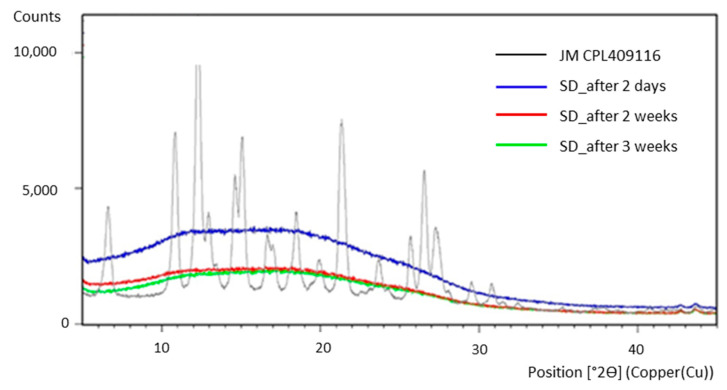
XRPD patterns of jet-milled CPL409116 (the black line shows the characteristic sharp peaks of the crystal structure) and spray-dried CPL409116 (the blue line represents the sample stored for 2 days at room temperature; the red line represents the sample stored for 2 weeks at room temperature; and the green line represents the sample stored for 3 weeks at room temperature).

The X-ray powder diffraction (XRPD) technique was utilized for the solid-state characterization of CPL409116 after both jet milling and spray drying. The results presented in Figure 4 show that this compound was spray-dried into an amorphous powder, which remained stable for three weeks of storage at room temperature.

The characteristic sharp peaks of the jet-milled particles (Figure 4, black line) indicated that the jet-milled particles were crystalline, highlighting a significant difference between the two engineering approaches employed.

#### 3.1.4. API Intrinsic Dissolution

Obtaining an amorphous form by spray drying CPL409116 prompted the authors to evaluate intrinsic dissolution, given that amorphous solid dispersions are known to enhance solubility, as reported in the literature [37].

**Figure 5:**
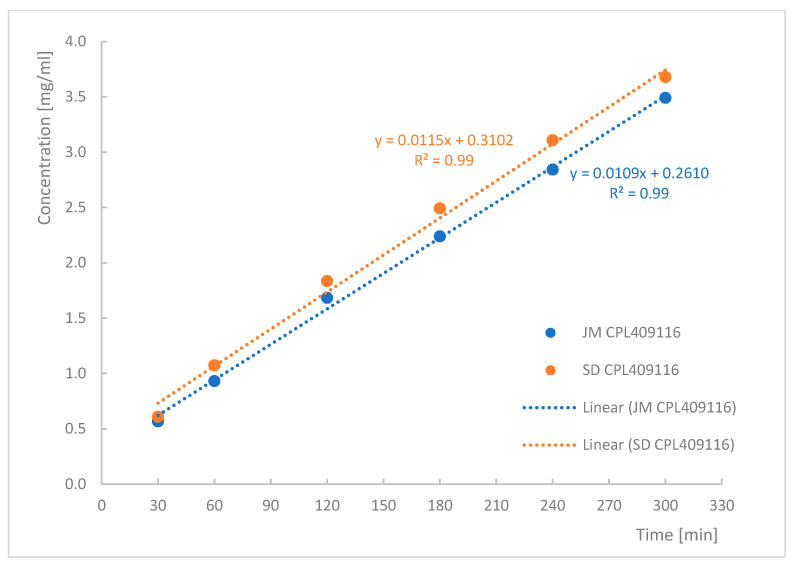
Intrinsic dissolution of jet-milled (blue) and spray-dried (orange) CPL409116.

The conducted analysis (Figure 5) revealed that the jet-milled CPL409116 dissolved in the chosen medium at a rate of 0.0109 mg/cm^2^/min, while the spray-dried sample dissolved at a rate of 0.0116 mg/cm^2^/min. 

The dissolution rates of both processed APIs were similar, indicating that the produced amorphous form did not improve the solubility of CPL409116. The similar dissolution rates of the JM and SD CPL409116 may be attributed to the fast crystallization phenomenon [38,39]. However, from a technological perspective, the SD process does not offer any advantages that would justify its selection as the primary method for obtaining APIs during the formulation development stage.

#### 3.1.5. Choosing a Technology for API Processing

The basis of both processes mentioned previously (particle collision in JM and the solution drying in SD) determined the formation of particles with entirely different shapes. High-energy impacts during jet milling enabled the production of a heterogeneous material with irregular particle shapes, whereas spray drying produced homogeneous particles with spherical shapes, differing only in diameter. 

Obtaining fine material as produced for compound CPL409116 after the JM process was related to the high energy generated during the process, which accumulated on the surface of the micronized particles. Despite the subsequent conditioning of the material after the process, interparticle interactions occurred, increasing cohesion. This is demonstrated in the SEM images (Figure 3), where the particles are shown to adhere to each other after the JM process, while SD allowed for a good separation of the particles. However, the issue of the high cohesion of the API particles may be resolved by adding a carrier to the formulation.

As noted by Hebbink et al. [21], each DPI formulation is unique and not easily explained by a single theory. Consequently, a specific development strategy is necessary for each new formulation. Therefore, assuming that the morphology of SD or JM CPL409116 particles would inherently improve product performance may not hold true. From a particle morphology standpoint, both processes are acceptable for inhaled formulations. Similarly, both processes yielded an acceptable PSD range. Hence, a solid-state form analysis, along with the intrinsic dissolution results, played a crucial role in selecting the leading technological approach for CPL409116 micronization.

According to the assumptions, the spray-drying process yielded an amorphous form of compound CPL409116 (Figure 4), which remained stable upon storage at a temperature of 22 ± 1 °C and a relative humidity of 23 ± 3% over the investigated period of three weeks. However, the intrinsic dissolution of the spray-dried API did not demonstrate a greater dissolution rate compared to the jet-milled CPL409116 (Figure 5). Therefore, jet milling was chosen as the primary technological process for CPL409116 processing to facilitate its inhalation delivery, due to its lower cost, higher process efficiency, and enhanced understanding. 

### 3.2. In Vitro Aerodynamic Properties

Carrier-based platforms always include lactose as a carrier and an active ingredient, with an optional force control agent (FCA) in the formulation that can perform various functions, including the following: improving the flowability and rheology, reducing interparticle adhesion, and enhancing particle deposition in the lungs. Additionally, sometimes the FCA reduces moisture effects and the impact on the chemical stability of the API [40,41,42].

The compounds are combined by either high-shear or low-shear mixing to ensure the uniformity of the active ingredient and to create formulation-specific interparticle interactions that are essential for the appropriate performance of the product.

Given that CPL409116 represents a first-in-class compound when administered via the inhalation route, its dose remains undetermined. Consequently, the following work aimed to identify factors that, if necessary, would allow the target FPF to be easily modified. The following factors were tested: the drug load, blender type, and force control agent. 

#### 3.2.1. Drug Load

According to the literature, higher drug loads in carrier-based formulations can lead to a decrease in the FPF values [43]. In the initial step of formulation development, an investigation was conducted to determine whether an increase in the percentage of the active ingredient would impact the FPF. Increasing the drug load from 7.5% to 30% resulted in a decrease in the FPF values from 48.8% to 37.0%. This outcome can be explained by the active sites theory: when the API concentration was 30%, all the adhesion sites on the surface of the lactose particles were filled by the particles of the active substance, leading to the formation of agglomerates with a low flowability. Therefore, increasing the API concentration in the developed carrier-based system will not lead to a higher FPF value.

Additionally, it was decided to perform stability testing for three months under long-term (25 °C; 60% RH) and accelerated (40 °C; 75% RH) conditions for both the blends discussed above, containing 7.5% and 30% API, respectively. The reported results showed that the level of related substances did not increase from the starting point through the testing period under any conditions and remained stable at 0.12% total impurities. This indicates that the investigated drug products will remain stable for three months in terms of related substances, according to European Pharmacopoeia guidelines.

#### 3.2.2. Blender Type

In the initial phase of this investigation, the content uniformity of the two blends produced by low-shear or high-shear mixing was evaluated. The low-shear blender, operating at its maximum speed (28 rpm for 30 min), yielded a mixing energy of 0.0004 mJ. In contrast, the high-shear blender, set to a medium speed within its available range (with a maximum value for TRV 1L of around 3000 rpm), achieved approximately 6.28 mJ in 3 min (Table 2).

The relative standard deviation (RSD) for the samples obtained from the low-shear blender was 11.55%, whereas for those from the high-shear blender, it was 0.35%. Despite operating at maximum speed, the low-shear blender did not achieve the desired homogeneity in the resulting mixture.

#### 3.2.3. Force Control Agent

The coating of lactose with magnesium stearate is widely recognized, and many drug products on the market contain this substance as a force control agent. Other substances, such as leucine, have been used for research purposes and are not included in carrier-based commercial formulations [44,45]. This excipient can be added in two ways. The first method (I) involves directly mixing the FCA with the carrier (which enables their surface interaction), followed by the addition of the active substance in the subsequent step. 

The second method (II) involves milling or micronizing the particles of both the active substance and the excipients (together with FCA) simultaneously. In this study, both methods were applied to prepare the blends containing CPL409116 (Table 3). 

For this purpose, a micronized lactose grade was used, eliminating the need for the further micronization of the lactose particles. Blend 1 was made using a high-shear blender by first mixing FCA with the carrier and then adding the previously jet-milled API, according to method I (Table 3, blend 1). Blend 3 was prepared according to method II: the previously jet-milled API was sieved twice with magnesium stearate through a 0.25 µm sieve, and then the powder was added to the lactose. All three substances were then mixed together in a high-shear blender (Table 3, blend 3). Blend 2 did not contain magnesium stearate, so both the carrier and the API that had previously undergone JM were mixed together using the high-shear blender. The resulting blends were analyzed using NGI at two flow rates—50 L/min and 100 L/min. The results are presented in Table 3. 

The FPF value for the blend without MgSt (blend 2) remained constant, regardless of the change in flow rate, and it was also the highest among all the blends. The lowest FPF was obtained for the mixture in which API was sieved with MgSt (blend 3). Additionally, for the blends containing MgSt (blend 1 and blend 3), a decrease in the flow rate resulted in a slight decrease in the FPF.

### 3.3. Quantitative Prediction of FPF Value

Based on the obtained in vitro results, a quantitative approach was applied to predict the FPF value and identify which variables in the formulated blends had the greatest influence on this value. 

The developed model took the form of the equation presented below:

FPF = Lactose_2_d10 − MgSt_perc^C [1] − tan(sin(sqrt(MgSt_perc*(Time_2 − Lactose_2_d10) + V_L_min − C [2]))) + C [3] − (−2*Lactose_2_d10 − 2*tan(API_percent) + C [4])/Lactose_1_d50 + C [5]*Time_2/API_percent,(3)

where C [1] = 1.0495; C [2] = 0.1175; C [3] = 248.6169; C [4] = 16753.1622; and C [5] = 3.8391.

**Figure 6:**
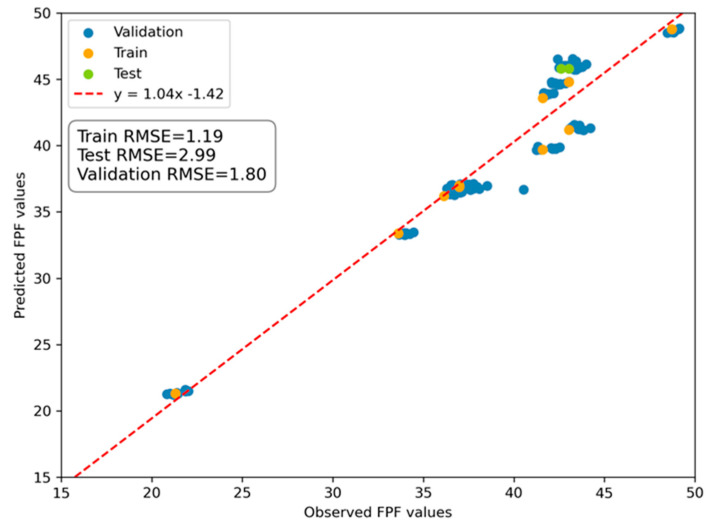
Predicted vs. observed FPF (fine particle fraction) values. The dashed red line represents the best fit for the validation data points. RMSE, root-mean-squared error.

The reported RMSEs for the training, testing, and validation datasets were 1.19, 2.99, and 1.80, respectively. The predicted vs. observed FPF values are presented in Figure 6.

**Figure 7:**
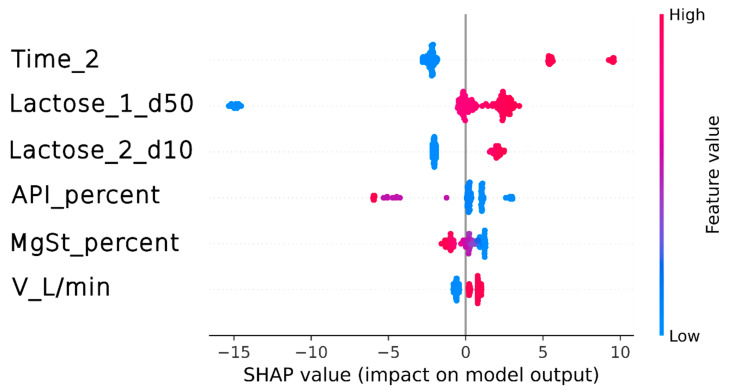
SHAP analysis results for the final model.

Based on the SHAP analysis of the developed model (Figure 7), the factors influencing the achieved FPF value included the following: the blending time of the active substance with lactose pre-mixed with MgSt (Time_2 [min]), the d50 of coarser lactose (Lactose_1_d50 [µm]), the d10 of finer lactose (Lactose_2_d10 [µm]), the percentage content of the active substance (API_percent [%]), the percentage content of magnesium stearate (MgSt_percent [%]), and, to a lesser extent, the flow rate (V_L/min).

The analysis of Figure 7 indicated that extending the blending time of the API with lactose pre-mixed with MgSt (Time_2 [min]) and increasing the d50 of coarser lactose (Lactose_1_d50 [µm]) or the d10 of finer lactose (Lactose_2_d10 [µm]) can positively affect the FPF by increasing its value by 2–10%. Conversely, reducing the blending time of the API with lactose pre-mixed with MgSt (Time_2 [min]) and reducing the d50 of coarser lactose (Lactose_1_d50 [µm]) or the d10 of finer lactose (Lactose_2_d10 [µm]) can lower the FPF by 2–15%.

In high-shear blenders, increasing the mixing time enhanced the FPF by increasing the ME (as demonstrated in Equation (2)). However, it is essential to note that this effect generally occurred within a specific range of ME values [24] and exceeding this range often resulted in a decrease in product performance. Therefore, in the next stages of the study, the optimal ME range for increasing the FPF should be determined experimentally.

In addition to the previously mentioned independent variables, the biggest decrease in the FPF (by about 7%) was associated with an increasing API concentration. As mentioned in Section 3.2.1, a reduction in the FPF values resulting from an increase in the active substance content of the formulation may be related to the active sites theory. Furthermore, this theory can coexist closely with the agglomerates theory [20]. According to these concepts, fine API particles bind to the active sites on coarser lactose particles. This process is saturable, and when all the active sites are covered, the remaining fine particles might form agglomerates, negatively affecting product performance [46,47].

Additionally, the content of magnesium stearate inversely affects the FPF. This influence can be attributed to the theory that an optimal percentage exists in lactose-based mixtures where its application is beneficial. Beyond this threshold, magnesium stearate tends to agglomerate, resulting in the coating that adversely affects the powder’s flow properties [48]. Although its impact is relatively minor compared to the variables previously mentioned, recognizing its influence on the FPF remains crucial because magnesium stearate can play roles beyond enhancing the performance of the target formulation, such as providing stabilization or acting as a water barrier [44].

Interestingly, the minimal influence of air flow on the FPF suggests that the performance of the formulation in delivering the API is independent of the flow rate, which could have important clinical implications, particularly for patients who may struggle with using dry powder formulations due to age-related or respiratory limitations (elderly, children) [49].

## 4. Conclusions

In light of the ongoing concern regarding pulmonary fibrosis in patients recovering from COVID-19, the development of effective therapies remains crucial. This study addressed this challenge by investigating a novel approach: the delivery of a potential treatment, CPL409116, a dual Janus and rho-associated kinase inhibitor, via dry powder inhalation.

The initial examination showed that both spray drying and jet milling produced particles primarily under 10 μm. Despite producing an amorphous form, spray drying did not significantly improve the dissolution rate compared to jet milling. Ultimately, jet milling was chosen for its lower cost, higher efficiency, and better process understanding.

It was shown that, in the carrier-based formulation, increasing the drug load from 7.5% to 30% reduced the FPF from 48.8% to 37.0% due to agglomeration. High-shear mixing produced more homogeneous blends compared to low-shear mixing and the addition of magnesium stearate (MgSt) slightly reduced the FPF but maintained consistency across different flow rates.

A predictive model identified the following key variables affecting the FPF: the blending time, the particle sizes of lactose, the API percentage, the MgSt percentage, and the flow rate. Extending the blending time and increasing the lactose particle sizes improved the FPF, while increasing the API concentration and the MgSt content slightly decreased it. Airflow had minimal impact on the FPF, which is important for patients with dosing challenges. Therefore, the key factors influencing the FPF in carrier-based formulations were identified, guiding future development strategies.

The results revealed that up to 50% of the compound is expected to reach the lower respiratory tract as fine particles, based on in vitro aerodynamic property studies. These studies focused on assessing the FPF rather than the distribution of the active substance across different stages of the impactor, as the distribution of receptors for the innovative compound CPL409116 in the lower respiratory tract remains unknown. Based on the authors’ experience, the achieved FPF value is sufficient for toxicological studies and for determining a safe and effective dose of CPL409116-containing dry powder for inhalation.

As shown, magnesium stearate may not be necessary due to the product’s performance, but further work will determine whether its use is required for the powder’s rheology during the encapsulation process or the sampling of the powder from the capsule and inhaler. Stability studies will indicate whether this CPL409116-containing formulation requires magnesium stearate as a water-barrier compound.

Future plans involve fine-tuning the carrier particle size distribution, adjusting the content of the active substance and magnesium stearate, and optimizing the mixing energy. There will be a need to identify which lactose fraction influences the FPF value and how it impacts the distribution of the active substance in the impactor stages. Additionally, after the appropriate lactose grade is selected, the ME range will be established for the chosen high-shear blender. This range will ensure content uniformity and enable the control of the FPF parameter within the specified limits.

## Figures and Tables

**Table 1:** Comparison of jet-milled and spray-dried CPL409116.

Parameter		Jet-Milled CPL409116	Spray-Dried CPL409116
Particle size distribution			
D(v. 0.1) [µm]		0.59	0.49
D(v. 0.5) [µm]		2.96	2.43
D(v. 0.9) [µm]		7.75	8.13
SPAN		2.42	3.14
Fraction under 10 µm [%]		96.05	94.15
Intrinsic dissolution			
Dissolved amount [mg/cm^2^·min]		0.0109	0.0116
Morphology			
Particle width	D(n. 0.5) [µm]	1.38	2.17
	D(n. 0.9) [µm]	3.02	4.52
Particle length	D(n. 0.5) [µm]	1.60	2.45
	D(n. 0.9) [µm]	4.13	5.67
Aspect ratio	D(n. 0.5) [µm]	0.87	0.92
	D(n. 0.9) [µm]	0.96	0.97
Circularity	D(n. 0.5) [µm]	0.96	0.97
	D(n. 0.9) [µm]	0.99	0.99
Elongation	D(n. 0.5) [µm]	0.13	0.08
	D(n. 0.9) [µm]	0.33	0.27
Powder density			
Bulk density [g/mL]		0.30	0.22
Tapped density [g/mL]		0.37	0.31
Carr’s index		18.92	29.03
Hausner ratio		1.23	1.41

**Table 2:** Blend content uniformity depending on blender type.

Blender Type	RPM	Mixing Time [min]	Mixing Energy [mJ]	Mean Recovery [%]	MIN [%]	MAX [%]	RSD
Low-shear	28	30	0.0004	104.05	96.18	134.47	11.55
High-shear	1500	3	6.28	96.14	95.70	96.70	0.35

**Table 3:** In vitro results for blends 1, 2, and 3.

No.	MgSt [%]	100 L/min			50 L/min		
		FPF [%]	MMAD [µm]	GSD	FPF [%]	MMAD [µm]	GSD
1	1	44.8	2.9	2.0	43.6	3.3	2.0
2	-	45.8	3.5	1.7	45.8	3.5	1.7
3	1	41.2	3.3	2.0	39.7	3.7	1.9

## Data Availability

The original contributions presented in the study are included in the article, further inquiries can be directed to the corresponding author.
